# Crystal structure of *N*′-[(*E*)-(1*S*,3*R*)-(3-isopropyl-1-methyl-2-oxo­cyclo­pent­yl)methyl­idene]-4-methyl­benzene­sulfono­hydrazide

**DOI:** 10.1107/S2056989015020307

**Published:** 2015-11-04

**Authors:** David Tymann, Dina Christina Dragon, Christopher Golz, Hans Preut, Carsten Strohmann, Martin Hiersemann

**Affiliations:** a Fakultät Chemie und Chemische Biologie, Technische Universität Dortmund, Otto-Hahn-Strasse 6, 44221 Dortmund, Germany

**Keywords:** crystal structure, hydrogen bonding, terpenoid synthesis

## Abstract

The title compound, C_17_H_24_N_2_O_3_S, was synthesized in order to determine the relative configuration of the corresponding β-keto aldehyde. In the U-shaped mol­ecule, the five-membered ring approximates an envelope, with the methyl­ene C atom adjacent to the quaternary C atom being the flap, and the methyl and isopropyl substituents lying to the same side of the ring. The dihedral angles between the four nearly coplanar atoms of the five-membered ring and the flap and the aromatic ring are 35.74 (15) and 55.72 (9)°, respectively. The bond angles around the S atom are in the range from 103.26 (12) to 120.65 (14)°. In the crystal, mol­ecules are linked *via* N—H⋯O hydrogen bonds, forming a chain along the *a* axis.

## Related literature   

For the synthesis of terpenoid-related buildings blocks, in particular cyclo­penta­noids, see: Helmboldt *et al.* (2006 ▸); Gille *et al.* (2011 ▸); Becker *et al.* (2013 ▸); Tymann *et al.* (2014 ▸). For the crystal structure of the corresponding *trans*-diastereomer, see: Tymann *et al.* (2015 ▸). For a review on cyclo­penta­noids by ring contraction, see: Silva (2002 ▸). For a solid-acid catalysed rearrangement of cyclic α,β-ep­oxy ketones, see: Elings *et al.* (2000 ▸).
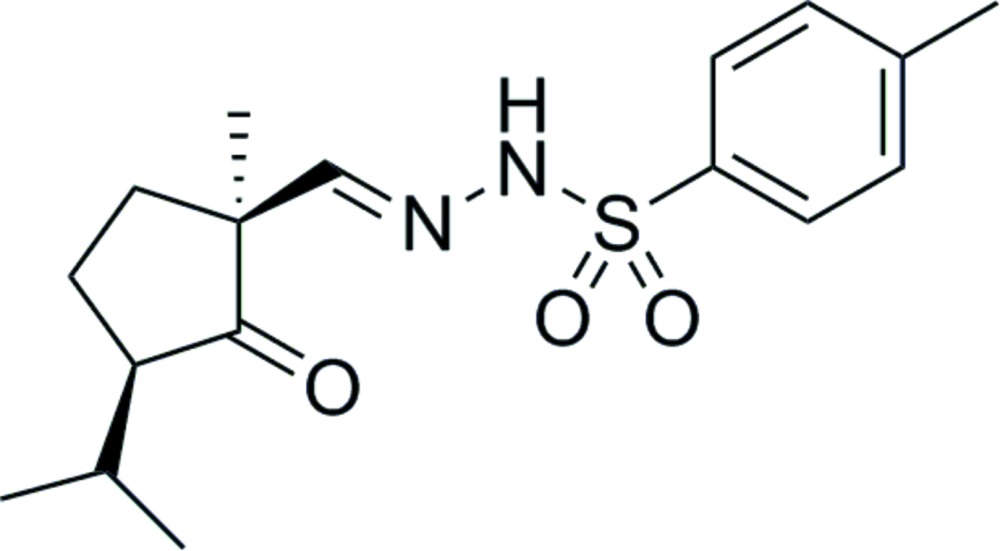


## Experimental   

### Crystal data  

C_17_H_24_N_2_O_3_S
*M* *_r_* = 336.44
Orthorhombic, 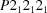

*a* = 9.4918 (7) Å
*b* = 13.2348 (12) Å
*c* = 14.6691 (12) Å
*V* = 1842.8 (3) Å^3^
*Z* = 4
Mo *K*α radiation
μ = 0.19 mm^−1^
*T* = 173 K
0.31 × 0.25 × 0.23 mm

### Data collection  

Oxford Diffraction Xcalibur Sapphire3 diffractometer
Absorption correction: multi-scan (*CrysAlis RED*; Oxford Diffraction, 2008 ▸) *T*_min_ = 0.98, *T*_max_ = 1.00
15093 measured reflections
4215 independent reflections
3526 reflections with *I* > 2σ(*I*)
*R*_int_ = 0.046

### Refinement  

*R*[*F* ^2^ > 2σ(*F* ^2^)] = 0.042
*wR*(*F* ^2^) = 0.093
*S* = 1.05
4215 reflections
216 parameters
H atoms treated by a mixture of independent and constrained refinement
Δρ_max_ = 0.22 e Å^−3^
Δρ_min_ = −0.33 e Å^−3^
Absolute structure: Flack *x* determined using 1277 quotients [(*I* ^+^)-(*I* ^-^)]/[(*I* ^+^)+(*I* ^-^)] (Parsons & Flack, 2004 ▸)
Absolute structure parameter: −0.02 (4)

### 

Data collection: *CrysAlis CCD* (Oxford Diffraction, 2008 ▸); cell refinement: *CrysAlis CCD*; data reduction: *CrysAlis CCD*; program(s) used to solve structure: *SHELXS97* (Sheldrick, 2008 ▸); program(s) used to refine structure: *SHELXL2013* (Sheldrick, 2015 ▸); molecular graphics: *SHELXTL-Plus* (Sheldrick, 2008 ▸); software used to prepare material for publication: *SHELXL97* (Sheldrick, 2008 ▸) and *PLATON* (Spek, 2009 ▸).

## Supplementary Material

Crystal structure: contains datablock(s) I, 2887. DOI: 10.1107/S2056989015020307/tk5399sup1.cif

Structure factors: contains datablock(s) I. DOI: 10.1107/S2056989015020307/tk5399Isup2.hkl

Supporting information file. DOI: 10.1107/S2056989015020307/tk5399Isup3.cml

**Fig. 1.**
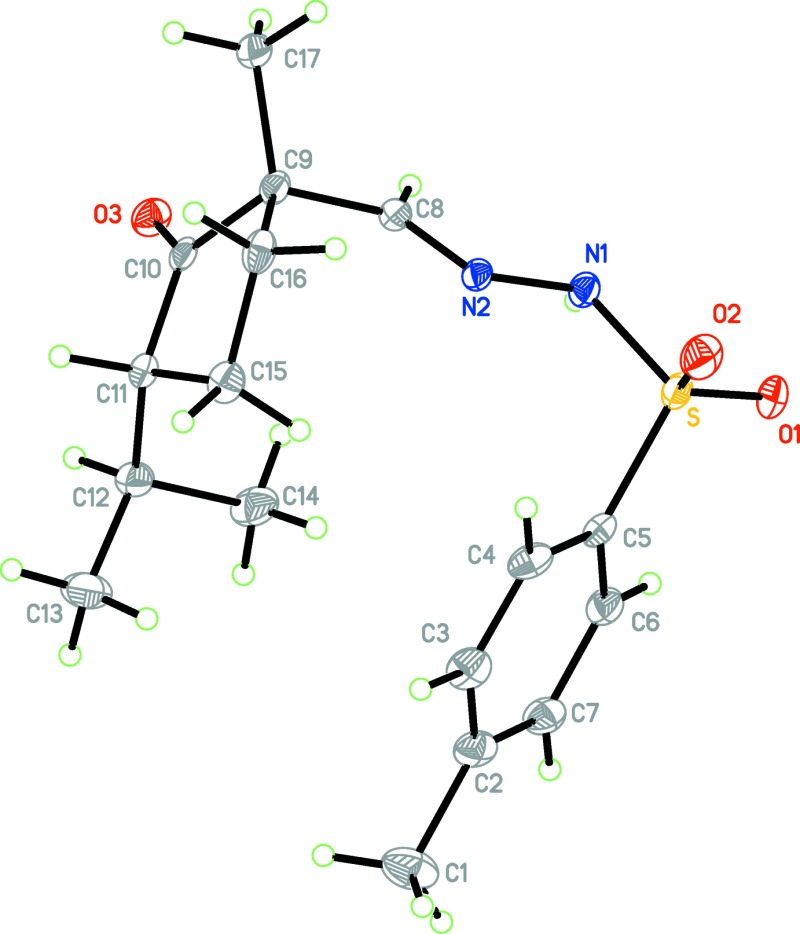
The molecular structure of the title compound, showing the labelling of all non-H atoms. Displacement ellipsoids are shown at the 50% probability level.

. DOI: 10.1107/S2056989015020307/tk5399fig1.tif
The mol­ecular structure of the title compound, showing the labelling of all non-H atoms. Displacement ellipsoids are shown at the 50% probability level.

CCDC reference: 1037859

Additional supporting information:  crystallographic information; 3D view; checkCIF report

## Figures and Tables

**Table 1:** Hydrogen-bond geometry (Å, °)

*D*—H⋯*A*	*D*—H	H⋯*A*	*D*⋯*A*	*D*—H⋯*A*
N1—H1*N*⋯O3^i^	0.86 (3)	2.00 (4)	2.836 (3)	164 (3)

Symmetry code: (i) 
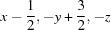
.

## supplementary crystallographic information

### S1. Comment

Prompted by our efforts in natural product synthesis, we seek access to cyclopentyl units. Herein, we chose a ring contraction strategy of a cyclic epoxy ketone. A Brønsted-acid promoted semi-pinacol rearrangement of *trans*-piperitone oxide afforded *cis*-3-isopropyl-1-methyl-2-oxocyclopentane-1-carbaldehyde (II) along with 2-hydroxypiperitone. A condensation of (II) with *p*-toulenesulfonyl hydrazide afforded the title compound, (I).

### S2. Experimental

A sealable glass pressure tube was charged with a solution of *cis*-3-isopropyl-1-methyl-2-oxocyclopentane-1-carbaldehyde (II) (C_10_H_16_O_2_, *M* = 168.23 g/mol, [α]_D_^20^ = -107.4 (c 0.059, CHCl_3_), 40 mg, 0.238 mmol, 1.0 eq) and *p*-toluenesulfonyl hydrazide (C_7_H_10_N_2_O_2_S, *M* = 186.23 g/mol, 62 mg, 0.333 mmol, 1.4 eq) in methanol (3 ml, 12.6 ml/mmol). The tube was sealed with a Teflon screw cap and stirred for 75 min at ambient temperature. Next, the reaction mixture was concentrated *in vacuo* and loaded onto silica gel with CH_2_Cl_2_ for flash chromatography (cyclohexane/ethyl acetate 10/1) to deliver the title compound (I) (C_17_H_24_N_2_O_3_S, *M* = 336.45 g/mol, 56 mg, 0.166 mmol, 70%) as a white solid and as an apparent mixture of double bond isomers (ratio = 95:5). Subsequent recrystallization of (I) from *n*-pentane/Et_2_O provided colourless crystals of the *E*-configured double bond isomer of (I). The ratio of isomers was determined by integration of the ^1^H NMR signals at 0.35 p.p.m. (d, *J* = 6.9 Hz, 3H) and 0.47 p.p.m. (d, *J* = 6.9 Hz, 3H). Characterization data are reported for the major compound from the mixture of double bond isomers. *R*_f_ 0.41 (cyclohexane/ethyl acetate 2/1); m.p. 388–391 K; ^1^H NMR (CDCl_3_, 500 MHz) δ 0.47 (d, *J* = 6.9 Hz, 3H), 0.85 (d, *J* = 6.9 Hz, 3H), 1.16 (s, 3H), 1.48–1.59 (m, 2H), 1.82–1.95 (m, 2H), 2.11 (td, *J* = 9.4 Hz, *J* = 5.0 Hz, 1H), 2.42 (s, 3H), 2.49–2.53 (m, 1H), 6.88 (s, 1H), 7.30 (d, *J* = 8.0 Hz, 2H), 7.78 (d, *J* = 8.0 Hz, 2H), 7.89 (s, 1H). ^13^C NMR (CDCl_3_, 126 MHz) δ 18.4 (CH_3_), 20.8 (CH_3_), 21.2 (CH_2_), 21.5 (CH_3_), 21.9 (CH_3_), 27.8 (CH), 31.6 (CH_2_), 53.8 (C), 54.7 (CH), 128.0 (2x CH), 129.6 (2x CH), 135.1 (C), 144.2 (C), 150.6 (CH), 219.1 (C). IR ν 3415 (w), 3126 (*s*), 2960 (*s*), 22870 (*s*), 1725 (*s*), 1600 (*m*), 1455 (*s*), 1360 (*s*), 1330 (*m*), 1170 (*s*), 1095 (*m*), 1050 (*s*), 940 (*m*) 820 (*s*); Anal. Calcd. for C_17_H_24_N_2_O_3_S: C, 60.7, H, 7.2, N, 8.3. Found: C, 60.6, H, 7.3, N, 8.3.

### S3. Refinement

H-atoms attached to C, except those in CH_3_, were placed in calculated positions (C—H = 0.95 - 1.00 Å and *U*_iso_(H) = 1.2 *U*_eq_(C)). All CH_3_ hydrogen atoms, which were taken from a Fourier map (AFIX 137), were allowed to rotate but not to tip (C—H = 0.98 Å and *U*_iso_(H) = 1.5 *U*_eq_(C)). The H-atom at N was taken from a Fourier map and refined isotropically.

### Crystal data

**Table:**

C_17_H_24_N_2_O_3_S	*D*_x_ = 1.213 Mg m^−^^3^
*M**_r_* = 336.44	Mo *K*α radiation, λ = 0.71073 Å
Orthorhombic, *P*2_1_2_1_2_1_	Cell parameters from 3585 reflections
*a* = 9.4918 (7) Å	θ = 2.6–27.1°
*b* = 13.2348 (12) Å	µ = 0.19 mm^−^^1^
*c* = 14.6691 (12) Å	*T* = 173 K
*V* = 1842.8 (3) Å^3^	Block, colourless
*Z* = 4	0.31 × 0.25 × 0.23 mm
*F*(000) = 720	

### Data collection

**Table:**

Oxford Diffraction Xcalibur Sapphire3 diffractometer	4215 independent reflections
Radiation source: Enhance (Mo) X-ray Source	3526 reflections with *I* > 2σ(*I*)
Detector resolution: 16.0560 pixels mm^-1^	*R*_int_ = 0.046
φ and ω scans	θ_max_ = 28.0°, θ_min_ = 2.6°
Absorption correction: multi-scan (*CrysAlis RED*; Oxford Diffraction, 2008)	*h* = −12→12
*T*_min_ = 0.98, *T*_max_ = 1.00	*k* = −15→16
15093 measured reflections	*l* = −19→18

### Refinement

**Table:**

Refinement on *F*^2^	Secondary atom site location: difference Fourier map
Least-squares matrix: full	Hydrogen site location: mixed
*R*[*F*^2^ > 2σ(*F*^2^)] = 0.042	H atoms treated by a mixture of independent and constrained refinement
*wR*(*F*^2^) = 0.093	*w* = 1/[σ^2^(*F*_o_^2^) + (0.0401*P*)^2^ + 0.1494*P*] where *P* = (*F*_o_^2^ + 2*F*_c_^2^)/3
*S* = 1.05	(Δ/σ)_max_ < 0.001
4215 reflections	Δρ_max_ = 0.22 e Å^−^^3^
216 parameters	Δρ_min_ = −0.33 e Å^−^^3^
0 restraints	Absolute structure: Flack *x* determined using 1277 quotients [(*I*^+^)-(*I*^-^)]/[(*I*^+^)+(*I*^-^)] (Parsons & Flack, 2004)
Primary atom site location: structure-invariant direct methods	Absolute structure parameter: −0.02 (4)

### Special details

**Table:**

Geometry. All e.s.d.'s (except the e.s.d. in the dihedral angle between two l.s. planes) are estimated using the full covariance matrix. The cell e.s.d.'s are taken into account individually in the estimation of e.s.d.'s in distances, angles and torsion angles; correlations between e.s.d.'s in cell parameters are only used when they are defined by crystal symmetry. An approximate (isotropic) treatment of cell e.s.d.'s is used for estimating e.s.d.'s involving l.s. planes.
Refinement. Refinement of *F*^2^ against ALL reflections. The weighted *R*-factor *wR* and goodness of fit *S* are based on *F*^2^, conventional *R*-factors *R* are based on *F*, with *F* set to zero for negative *F*^2^. The threshold expression of *F*^2^ > σ(*F*^2^) is used only for calculating *R*-factors(gt) *etc*. and is not relevant to the choice of reflections for refinement. *R*-factors based on *F*^2^ are statistically about twice as large as those based on *F*, and *R*-factors based on ALL data will be even larger.

### Fractional atomic coordinates and isotropic or equivalent isotropic displacement parameters (Å^2^)

**Table:**

	*x*	*y*	*z*	*U*_iso_*/*U*_eq_	
S	0.57466 (7)	0.53116 (6)	0.08855 (5)	0.02777 (17)	
O1	0.45052 (19)	0.57619 (18)	0.05053 (14)	0.0389 (6)	
O2	0.5837 (2)	0.42449 (16)	0.10068 (15)	0.0411 (5)	
O3	1.1776 (2)	0.72234 (15)	−0.00066 (13)	0.0309 (5)	
N1	0.6994 (2)	0.5647 (2)	0.01656 (15)	0.0241 (5)	
N2	0.8352 (2)	0.53514 (19)	0.04375 (14)	0.0226 (5)	
C1	0.7064 (4)	0.7294 (3)	0.4507 (2)	0.0510 (10)	
H1A	0.6958	0.6789	0.4990	0.077*	
H1B	0.6409	0.7854	0.4614	0.077*	
H1C	0.8033	0.7550	0.4506	0.077*	
C2	0.6745 (3)	0.6814 (2)	0.35949 (19)	0.0333 (7)	
C3	0.7286 (3)	0.5861 (3)	0.3378 (2)	0.0358 (8)	
H3	0.7880	0.5526	0.3803	0.043*	
C4	0.6978 (3)	0.5398 (2)	0.25650 (18)	0.0308 (6)	
H4	0.7341	0.4745	0.2433	0.037*	
C5	0.6127 (3)	0.5896 (2)	0.19358 (18)	0.0259 (6)	
C6	0.5606 (3)	0.6853 (2)	0.21212 (19)	0.0303 (7)	
H6	0.5052	0.7199	0.1681	0.036*	
C7	0.5902 (3)	0.7302 (2)	0.2954 (2)	0.0329 (7)	
H7	0.5526	0.7950	0.3089	0.039*	
C8	0.9350 (3)	0.5757 (2)	−0.00108 (18)	0.0240 (6)	
H8	0.9128	0.6228	−0.0478	0.029*	
C9	1.0880 (3)	0.5513 (2)	0.01786 (18)	0.0251 (6)	
C10	1.1497 (3)	0.6526 (2)	0.05022 (18)	0.0227 (6)	
C11	1.1652 (3)	0.6526 (2)	0.15308 (17)	0.0247 (6)	
H11	1.2680	0.6440	0.1659	0.030*	
C12	1.1208 (3)	0.7517 (2)	0.1995 (2)	0.0320 (7)	
H12	1.1814	0.8067	0.1739	0.038*	
C13	1.1488 (4)	0.7473 (3)	0.3020 (2)	0.0475 (9)	
H13A	1.0847	0.6984	0.3302	0.071*	
H13B	1.1332	0.8142	0.3288	0.071*	
H13C	1.2464	0.7264	0.3127	0.071*	
C14	0.9673 (3)	0.7800 (3)	0.1793 (2)	0.0416 (8)	
H14A	0.9047	0.7275	0.2034	0.062*	
H14B	0.9538	0.7858	0.1133	0.062*	
H14C	0.9451	0.8448	0.2084	0.062*	
C15	1.0931 (3)	0.5542 (2)	0.18366 (19)	0.0306 (7)	
H15A	1.1406	0.5254	0.2379	0.037*	
H15B	0.9927	0.5661	0.1984	0.037*	
C16	1.1078 (3)	0.4835 (2)	0.1014 (2)	0.0304 (7)	
H16A	1.2019	0.4512	0.1006	0.036*	
H16B	1.0349	0.4300	0.1030	0.036*	
C17	1.1573 (3)	0.5148 (2)	−0.0706 (2)	0.0359 (7)	
H17A	1.2582	0.5039	−0.0602	0.054*	
H17B	1.1447	0.5660	−0.1183	0.054*	
H17C	1.1135	0.4513	−0.0900	0.054*	
H1N	0.690 (3)	0.627 (3)	0.001 (2)	0.040 (10)*	

### Atomic displacement parameters (Å^2^)

**Table:**

	*U*^11^	*U*^22^	*U*^33^	*U*^12^	*U*^13^	*U*^23^
S	0.0189 (3)	0.0290 (4)	0.0354 (4)	−0.0034 (3)	0.0053 (3)	−0.0060 (3)
O1	0.0171 (10)	0.0542 (15)	0.0455 (12)	0.0008 (10)	−0.0003 (9)	−0.0093 (11)
O2	0.0389 (11)	0.0293 (12)	0.0549 (14)	−0.0106 (10)	0.0121 (11)	−0.0049 (10)
O3	0.0323 (10)	0.0271 (12)	0.0334 (11)	−0.0043 (9)	0.0072 (9)	0.0008 (10)
N1	0.0182 (11)	0.0256 (14)	0.0286 (13)	0.0020 (10)	0.0006 (9)	−0.0015 (10)
N2	0.0177 (10)	0.0213 (12)	0.0286 (11)	0.0023 (10)	0.0013 (9)	−0.0062 (10)
C1	0.077 (3)	0.047 (2)	0.0290 (17)	−0.009 (2)	0.0018 (17)	0.0017 (16)
C2	0.0404 (17)	0.0341 (19)	0.0255 (15)	−0.0055 (15)	0.0097 (13)	0.0052 (12)
C3	0.0395 (17)	0.039 (2)	0.0291 (16)	0.0024 (15)	0.0036 (13)	0.0138 (14)
C4	0.0358 (15)	0.0263 (16)	0.0302 (15)	0.0065 (14)	0.0113 (12)	0.0071 (13)
C5	0.0204 (13)	0.0296 (17)	0.0278 (14)	−0.0006 (11)	0.0080 (11)	0.0012 (12)
C6	0.0249 (15)	0.0327 (17)	0.0334 (16)	0.0049 (14)	0.0025 (12)	−0.0005 (12)
C7	0.0352 (16)	0.0260 (17)	0.0375 (16)	0.0031 (14)	0.0084 (14)	−0.0025 (13)
C8	0.0219 (13)	0.0253 (15)	0.0247 (13)	−0.0015 (12)	0.0019 (11)	−0.0030 (11)
C9	0.0180 (12)	0.0243 (16)	0.0329 (14)	−0.0006 (12)	0.0038 (11)	−0.0059 (11)
C10	0.0126 (12)	0.0245 (16)	0.0310 (14)	0.0022 (11)	0.0036 (11)	−0.0035 (12)
C11	0.0159 (12)	0.0303 (17)	0.0279 (14)	0.0000 (12)	0.0005 (11)	−0.0016 (12)
C12	0.0323 (16)	0.0319 (18)	0.0319 (16)	−0.0016 (13)	0.0042 (12)	−0.0074 (13)
C13	0.054 (2)	0.057 (3)	0.0313 (17)	0.0063 (18)	0.0011 (16)	−0.0120 (16)
C14	0.0392 (18)	0.042 (2)	0.0434 (19)	0.0141 (16)	0.0068 (15)	−0.0046 (15)
C15	0.0272 (14)	0.0316 (17)	0.0330 (15)	0.0023 (13)	−0.0008 (12)	0.0076 (12)
C16	0.0210 (13)	0.0226 (16)	0.0475 (18)	0.0017 (11)	0.0010 (12)	0.0028 (13)
C17	0.0256 (14)	0.041 (2)	0.0417 (17)	−0.0039 (14)	0.0063 (13)	−0.0178 (14)

### Geometric parameters (Å, º)

**Table:**

S—O2	1.426 (2)	C9—C16	1.531 (4)
S—O1	1.433 (2)	C9—C17	1.534 (4)
S—N1	1.648 (2)	C9—C10	1.538 (4)
S—C5	1.761 (3)	C10—C11	1.516 (4)
O3—C10	1.216 (3)	C11—C12	1.537 (4)
N1—N2	1.405 (3)	C11—C15	1.539 (4)
N1—H1N	0.86 (3)	C11—H11	1.0000
N2—C8	1.272 (3)	C12—C13	1.528 (4)
C1—C2	1.511 (4)	C12—C14	1.533 (4)
C1—H1A	0.9800	C12—H12	1.0000
C1—H1B	0.9800	C13—H13A	0.9800
C1—H1C	0.9800	C13—H13B	0.9800
C2—C7	1.393 (4)	C13—H13C	0.9800
C2—C3	1.399 (5)	C14—H14A	0.9800
C3—C4	1.372 (4)	C14—H14B	0.9800
C3—H3	0.9500	C14—H14C	0.9800
C4—C5	1.392 (4)	C15—C16	1.533 (4)
C4—H4	0.9500	C15—H15A	0.9900
C5—C6	1.387 (4)	C15—H15B	0.9900
C6—C7	1.387 (4)	C16—H16A	0.9900
C6—H6	0.9500	C16—H16B	0.9900
C7—H7	0.9500	C17—H17A	0.9800
C8—C9	1.514 (3)	C17—H17B	0.9800
C8—H8	0.9500	C17—H17C	0.9800
			
O2—S—O1	120.65 (14)	O3—C10—C9	123.7 (2)
O2—S—N1	107.67 (13)	C11—C10—C9	110.1 (2)
O1—S—N1	103.26 (12)	C10—C11—C12	114.4 (2)
O2—S—C5	108.25 (14)	C10—C11—C15	104.3 (2)
O1—S—C5	109.04 (13)	C12—C11—C15	118.2 (2)
N1—S—C5	107.17 (12)	C10—C11—H11	106.4
N2—N1—S	113.73 (18)	C12—C11—H11	106.4
N2—N1—H1N	116 (2)	C15—C11—H11	106.4
S—N1—H1N	111 (2)	C13—C12—C14	111.3 (3)
C8—N2—N1	114.7 (2)	C13—C12—C11	110.8 (3)
C2—C1—H1A	109.5	C14—C12—C11	112.6 (3)
C2—C1—H1B	109.5	C13—C12—H12	107.3
H1A—C1—H1B	109.5	C14—C12—H12	107.3
C2—C1—H1C	109.5	C11—C12—H12	107.3
H1A—C1—H1C	109.5	C12—C13—H13A	109.5
H1B—C1—H1C	109.5	C12—C13—H13B	109.5
C7—C2—C3	118.4 (3)	H13A—C13—H13B	109.5
C7—C2—C1	121.2 (3)	C12—C13—H13C	109.5
C3—C2—C1	120.5 (3)	H13A—C13—H13C	109.5
C4—C3—C2	121.5 (3)	H13B—C13—H13C	109.5
C4—C3—H3	119.3	C12—C14—H14A	109.5
C2—C3—H3	119.3	C12—C14—H14B	109.5
C3—C4—C5	119.2 (3)	H14A—C14—H14B	109.5
C3—C4—H4	120.4	C12—C14—H14C	109.5
C5—C4—H4	120.4	H14A—C14—H14C	109.5
C6—C5—C4	120.6 (3)	H14B—C14—H14C	109.5
C6—C5—S	120.0 (2)	C16—C15—C11	104.3 (2)
C4—C5—S	119.4 (2)	C16—C15—H15A	110.9
C5—C6—C7	119.5 (3)	C11—C15—H15A	110.9
C5—C6—H6	120.3	C16—C15—H15B	110.9
C7—C6—H6	120.3	C11—C15—H15B	110.9
C6—C7—C2	120.8 (3)	H15A—C15—H15B	108.9
C6—C7—H7	119.6	C9—C16—C15	105.1 (2)
C2—C7—H7	119.6	C9—C16—H16A	110.7
N2—C8—C9	122.0 (3)	C15—C16—H16A	110.7
N2—C8—H8	119.0	C9—C16—H16B	110.7
C9—C8—H8	119.0	C15—C16—H16B	110.7
C8—C9—C16	112.9 (2)	H16A—C16—H16B	108.8
C8—C9—C17	108.9 (2)	C9—C17—H17A	109.5
C16—C9—C17	116.1 (2)	C9—C17—H17B	109.5
C8—C9—C10	103.6 (2)	H17A—C17—H17B	109.5
C16—C9—C10	102.6 (2)	C9—C17—H17C	109.5
C17—C9—C10	111.9 (2)	H17A—C17—H17C	109.5
O3—C10—C11	126.2 (3)	H17B—C17—H17C	109.5
			
O2—S—N1—N2	−55.9 (2)	N2—C8—C9—C17	124.3 (3)
O1—S—N1—N2	175.41 (19)	N2—C8—C9—C10	−116.4 (3)
C5—S—N1—N2	60.3 (2)	C8—C9—C10—O3	−76.0 (3)
S—N1—N2—C8	−167.6 (2)	C16—C9—C10—O3	166.3 (2)
C7—C2—C3—C4	−1.5 (4)	C17—C9—C10—O3	41.2 (4)
C1—C2—C3—C4	178.1 (3)	C8—C9—C10—C11	102.3 (2)
C2—C3—C4—C5	1.1 (4)	C16—C9—C10—C11	−15.4 (3)
C3—C4—C5—C6	0.7 (4)	C17—C9—C10—C11	−140.5 (2)
C3—C4—C5—S	179.2 (2)	O3—C10—C11—C12	40.3 (4)
O2—S—C5—C6	−155.0 (2)	C9—C10—C11—C12	−137.9 (2)
O1—S—C5—C6	−22.0 (3)	O3—C10—C11—C15	171.0 (3)
N1—S—C5—C6	89.2 (2)	C9—C10—C11—C15	−7.3 (3)
O2—S—C5—C4	26.5 (2)	C10—C11—C12—C13	−176.4 (2)
O1—S—C5—C4	159.5 (2)	C15—C11—C12—C13	60.2 (3)
N1—S—C5—C4	−89.3 (2)	C10—C11—C12—C14	58.2 (3)
C4—C5—C6—C7	−2.1 (4)	C15—C11—C12—C14	−65.2 (3)
S—C5—C6—C7	179.4 (2)	C10—C11—C15—C16	27.2 (3)
C5—C6—C7—C2	1.7 (4)	C12—C11—C15—C16	155.5 (2)
C3—C2—C7—C6	0.0 (4)	C8—C9—C16—C15	−78.7 (3)
C1—C2—C7—C6	−179.6 (3)	C17—C9—C16—C15	154.5 (2)
N1—N2—C8—C9	−179.0 (2)	C10—C9—C16—C15	32.2 (2)
N2—C8—C9—C16	−6.2 (4)	C11—C15—C16—C9	−37.7 (3)

### Hydrogen-bond geometry (Å, º)

**Table:**

*D*—H···*A*	*D*—H	H···*A*	*D*···*A*	*D*—H···*A*
N1—H1*N*···O3^i^	0.86 (3)	2.00 (4)	2.836 (3)	164 (3)

Symmetry code: (i) *x*−1/2, −*y*+3/2, −*z*.

## References

[bb1] Becker, J., Butt, L., von Kiedrowski, V., Mischler, E., Quentin, F. & Hiersemann, M. (2013). *Org. Lett.* **15**, 5982–5985. 10.1021/ol4028418 24215353

[bb2] Elings, J. A., Lempers, H. B. & Sheldon, R. A. (2000). *Eur. J. Org. Chem.* pp. 1905–1911.

[bb3] Gille, A., Rehbein, J. & Hiersemann, M. (2011). *Org. Lett.* **13**, 2122–2125. 10.1021/ol200558j 21428300

[bb4] Helmboldt, H., Köhler, D. & Hiersemann, M. (2006). *Org. Lett.* **8**, 1573–1576. 10.1021/ol060115t 16597113

[bb5] Oxford Diffraction (2008). *CrysAlis CCD* and *CrysAlis RED*. Oxford Diffraction Ltd, Yarnton, Oxfordshire, England.

[bb6] Parsons, S. & Flack, H. (2004). *Acta Cryst.* A**60**, s61.

[bb7] Sheldrick, G. M. (2008). *Acta Cryst.* A**64**, 112–122. 10.1107/S0108767307043930 18156677

[bb8] Sheldrick, G. M. (2015). *Acta Cryst.* C**71**, 3–8.

[bb9] Silva, L. F. Jr (2002). *Tetrahedron*, **58**, 9137–9161.

[bb10] Spek, A. L. (2009). *Acta Cryst.* D**65**, 148–155. 10.1107/S090744490804362X PMC2631630 19171970

[bb11] Tymann, D., Dragon, D. C., Golz, C., Preut, H., Strohmann, C. & Hiersemann, M. (2015). *Acta Cryst.* E**71**, o99–o100. 10.1107/S2056989014026747 PMC4384629 25878892

[bb12] Tymann, D., Klüppel, A., Hiller, W. & Hiersemann, M. (2014). *Org. Lett.* **16**, 4062–4065. 10.1021/ol501204m 25083751

