# Supplementary figures and images for: Crystal structure of N′-[(E)-(1S,3R)-(3-isopropyl-1-methyl-2-oxo­cyclo­pent­yl)methyl­idene]-4-methyl­benzene­sulfono­hydrazide
Source: Acta Crystallogr E Crystallogr Commun. 2015 Nov 4;71(Pt 12):o904–5. doi: 10.1107/S2056989015020307 (PMC4719872; doi:10.1107/S2056989015020307)

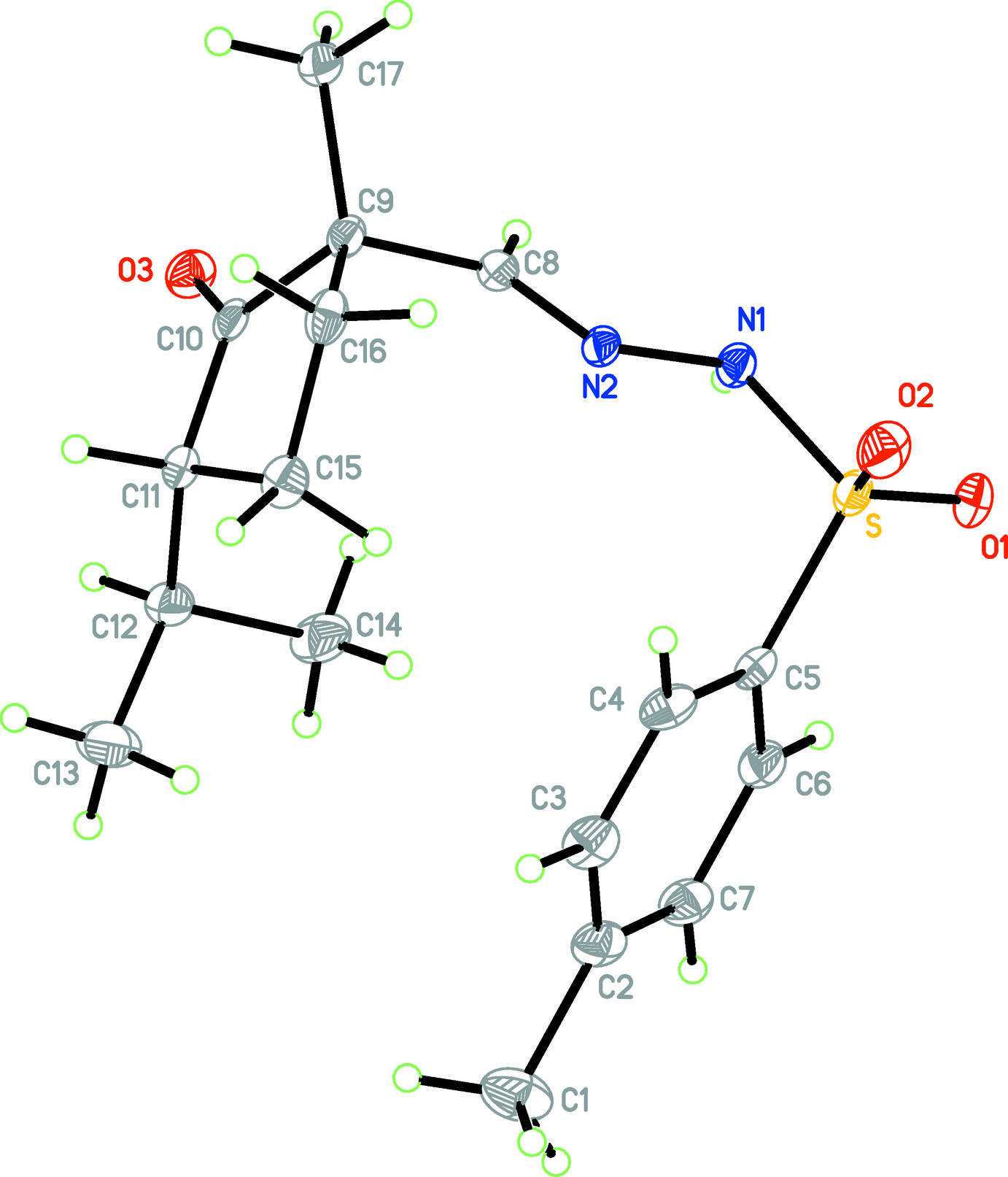

Supplement: Supplementary file 4 [file e-71-0o904-fig1.tif]
